# Middle Ear Papilloma

**DOI:** 10.1016/S1808-8694(15)30092-6

**Published:** 2015-10-19

**Authors:** Sandro de Menezes Santos Torres, Thomas Wagner Castro, Ricardo Ferreira Bento, Hélio Andrade Lessa

**Affiliations:** a SBORL Specialist, Substitute Professor of the Otorhinolaryngology Residency at UFBA, Advisor for Otorhinolaryngology Residents at the UFBA Medical School; b MD. Otorhinolaryngology Resident at HC-UFBA; c Member of the ABORL-CCF, Assistant Professor of Otorhinolaryngology at the Medical School of the University of São Paulo; d Member of the ABORL-CCF, Associate Professor at the HC-UFBA Medical School, Head of the Otorhinolaryngology Service at HC-UFBA. Otorhinolaryngology Service HUPES-HC-UFBA at the UFBA Medical School

**Keywords:** papilloma middle ear

## INTRODUCTION

Papillomas are benign, however locally invasive tumors normally originated in the larynx and nasosinusal tract^1^. These tumors can be described from their specific histological and morphologic characteristics. Recurrence is common if excision is inadequately performed. Tumors with similar histological and biological characteristics may occur less frequently in areas outside the nasosinusal tract such as pharynx, lacrimal sac and middle ear^2^. Middle ear and mastoid involvement is explained by two mechanisms: (1) direct extension from the nasosinusal cavity via the eustachian tube or (2) primary involvement of the middle ear secondary to metaplastic changes to the mucosal lining^3^.

As these tumors seldom involve the middle ear, there are no estimates on the incidence of these lesions in this particular site and only a few reports in the literature^4^, ^5^. This paper aims to report a case of primary middle ear papilloma in a patient with no account of previous nasal disease who evolved to facial palsy. The patient underwent radical mastoidectomy and has been asymptomatic after six months of follow-up.

## CASE REPORT

J.P.A., female, 27 years of age, Caucasian, born in Salvador, went to the otolaryngology service of the Professor Edgard Santos Hospital at the Federal University of Bahia complaining she has had persistent right-side otorrhea for the past 12 years associated to hypoacusis and ipsilateral humming. Otalgia, otorrhagia, and vertigo were not present. She evolved to total right-side facial palsy about 10 years ago, a condition still present at the time of surgery. No references were made to nasal or oropharyngeal problems.

Otoscopic findings revealed no alterations in the left ear, whereas the right ear presented ample central perforation of the tympanic membrane, retraction pouch in the attic and granulous tissue in the middle ear mucosa. The patient did not present right eye closure or ipsilateral lip commissure. Examination of the nose and oropharynx did not show noteworthy manifestations.

**Figure 1:**
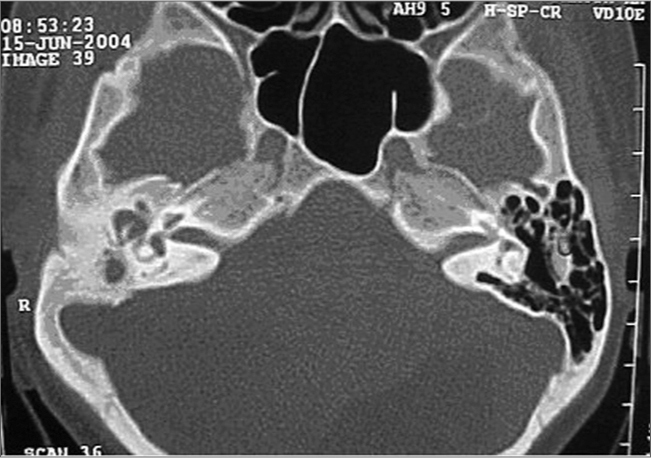
Middle ear papilloma - Post-op CT scan

Tonal and vocal audiometric tests were performed on August 16, 2004. The patient’s left ear showed no alterations, while the right ear displayed deep mixed auditory loss. A CT scan of the temporal bones (Figure 1) done on June 15, 2004 showed signs of an opaque middle ear cavity and mastoid cells to the right with no evidence of aggressive disease (osteolytic lesion).

The patient also underwent nasal examination with a 30-degree rigid endoscope and no evidence of nasal papilloma or any other relevant alterations were found.

The patient underwent right-side radical mastoidectomy on August 21, 2004. During the procedure brittle tissue involving the mastoid was removed, fascia of the temporal muscle placed over the remaining cavity, and broad meatoplasty performed.

The pathologist’s report (Sep. 20, 2004) indicated the presence of papilloma with hyperkeratosis.

The patient developed right-side intermittent otorrhea only for the first two months of post-op follow-up. Marked improvement on facial palsy was reported, mainly in relation to right-side eye closure, however little change was noticed on the lip commissure. Right ear otoscopic examination identified broad meatoplasty and a dry, fully epithelized mastoid cavity, satisfactory graft take and no crusts.

## DISCUSSION

Papillomas are benign epithelial tumors that involve the nasosinusal tract and may be grouped into three main categories: (1) inverted (endophytic), (2) cylindrical and (3) fungiform (exophytic). They may relapse when not properly excised and, in a few rare occasions, might evolve towards malignancy in the form of non-keratinized carcinomas. Therefore the tumor must be completely visualized and fully resected to thus mitigate recurrence.

The pathogenesis of papillomas remains controversial. Several authors have tried to correlate its etiology to tumor, viral, inflammatory, allergic, and environmental factors. Human papillomavirus (HPV) types 6 and 11 have been found in association with nasosinusal tract papilloma. Although no causal relationship has been established between the presence of HPV and the onset of papillomas, this is yet a possibility that requires strong consideration. It is more likely that the development of papillomas does not derive from one single factor such as HPV infection, but rather from the confluence of several factors. Nasosinusal tract papillomas may propagate towards the middle ear via Eustachian tube. This is, however, a remote possibility, especially when there are no nasopharyngeal papillomas colonizing the Eustachian tube. This concept is supported by Wenig^2^, Kaddour and Woodhead4 and also by us, as we have not been able to find any nasosinusal tract papillomas before, or co-existing with middle ear papillomas, a fact that has led us to consider a multicentric, primary origin for this disease.

Although rare, papillomas may occur outside the nasosinusal tract, in areas such as the nasopharynx, lacrimal sac and middle ear. Only very few cases of middle ear involvement have been reported. Wenig^2^ reported on a series of five patients diagnosed with middle ear papilloma. All patients were females with reported symptoms on the left ear. The most frequent complaint was reduced auditory acuity in the involved ear. All shared previous or concomitant history of chronic otitis media in agreement with the case reported here, although none of their patients presented facial palsy.

Primary differential diagnosis is done with middle ear adenoma. Differently from adenomas, papillomas are not a disease of glandular pattern, nor do they show the cytomorphologic characteristics seen in adenomas. Reactivity to immunohistochemistry with epithelial markers such as cytokeratin cannot differentiate papillomas from adenomas, as both are cytokeratin-reactive. Other middle ear tumors such as jugulotympanic paragangliomas, acoustic neuromas, and meningiomas do not pose diagnostic confusion with papilloma as they present specific histomorphologic characteristics.

The treatment for papillomas is eminently surgical, and tympanomastoidectomy is the procedure of choice. More conservative procedures such as myringectomy and simple excision were proven ineffective in controlling the disease, as reported by Wenig^2^. As recurrence rates may be high for this disease, shown by Wenig, radical mastoidectomy must be considered as the initial approach, as done in the case reported in this paper.
